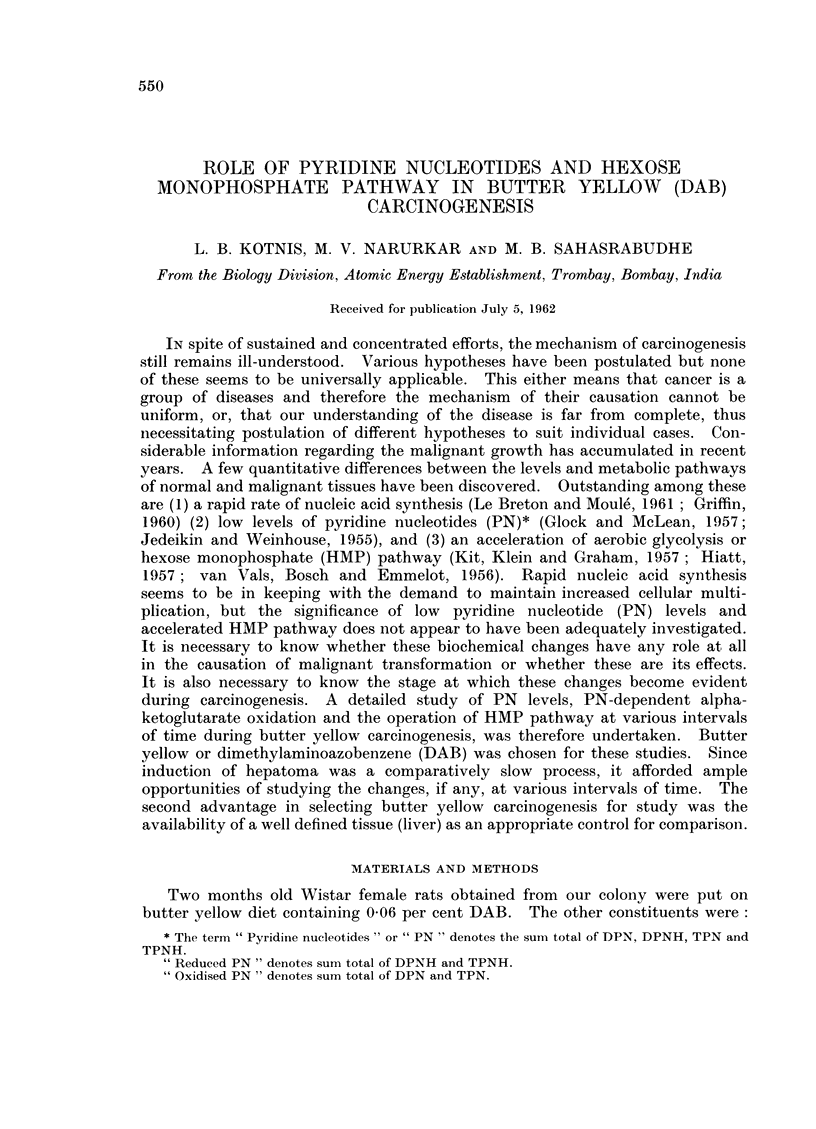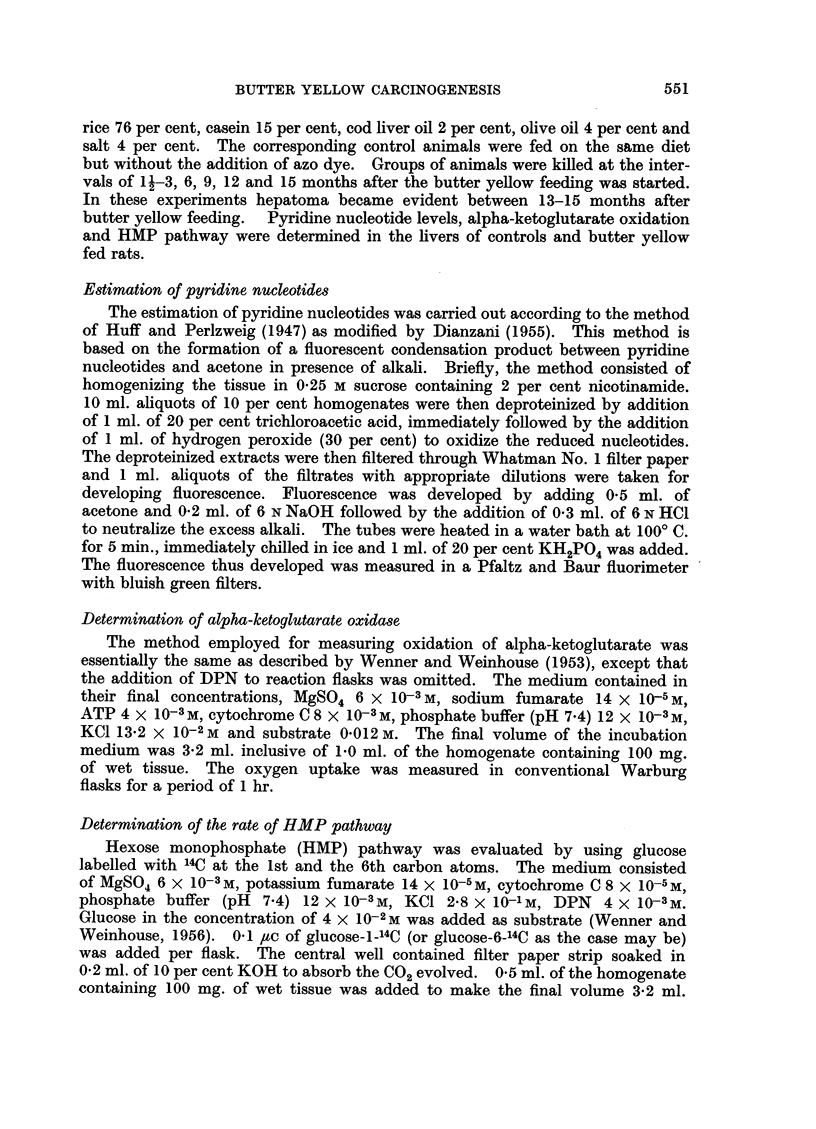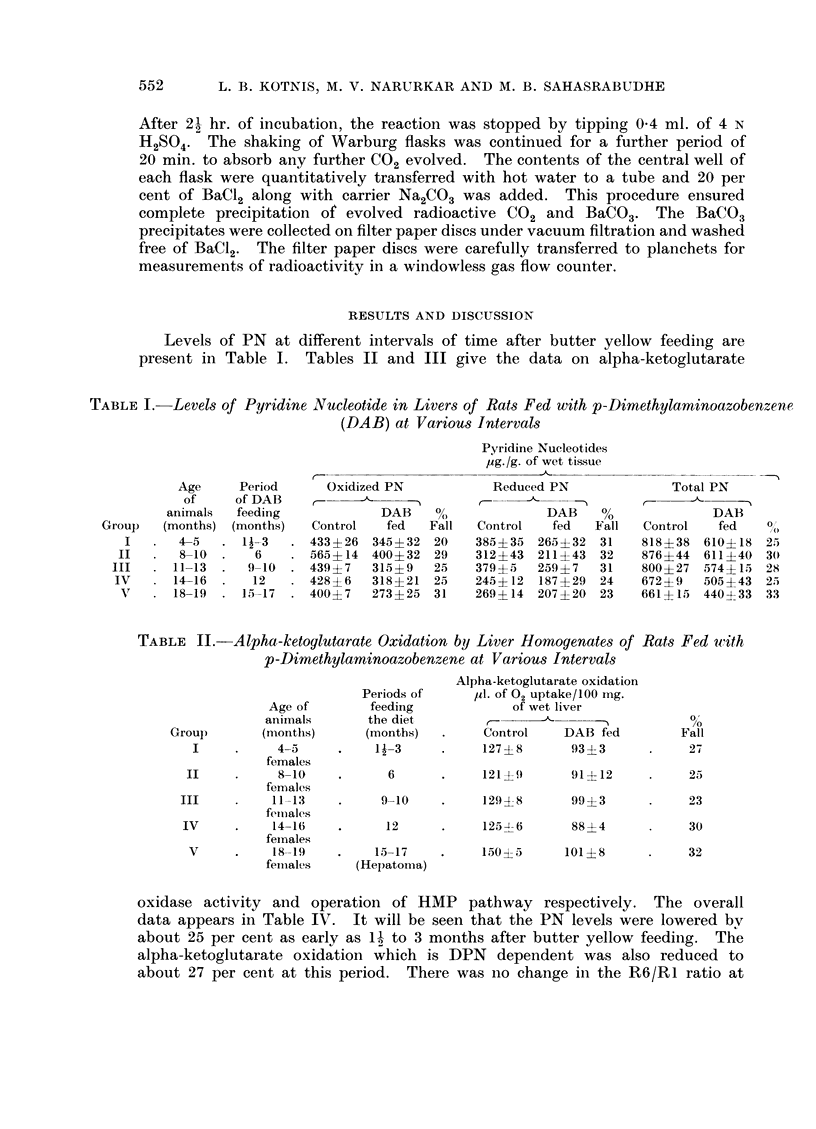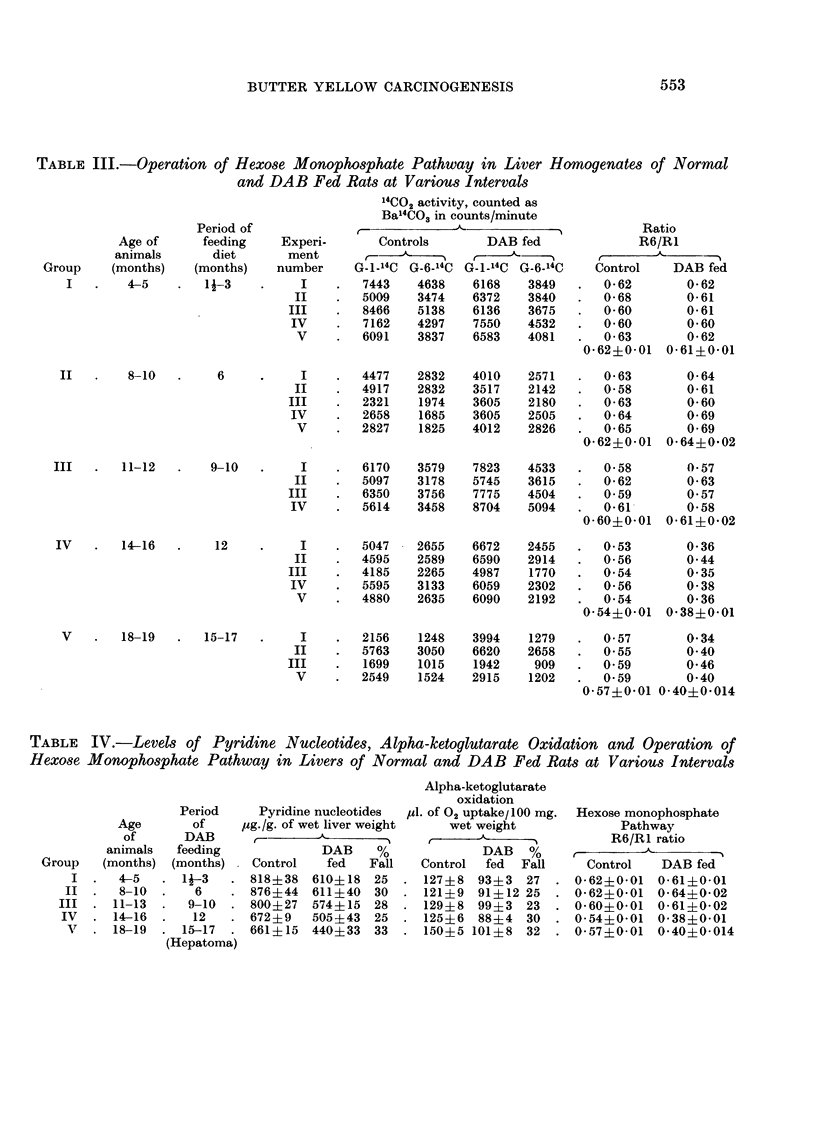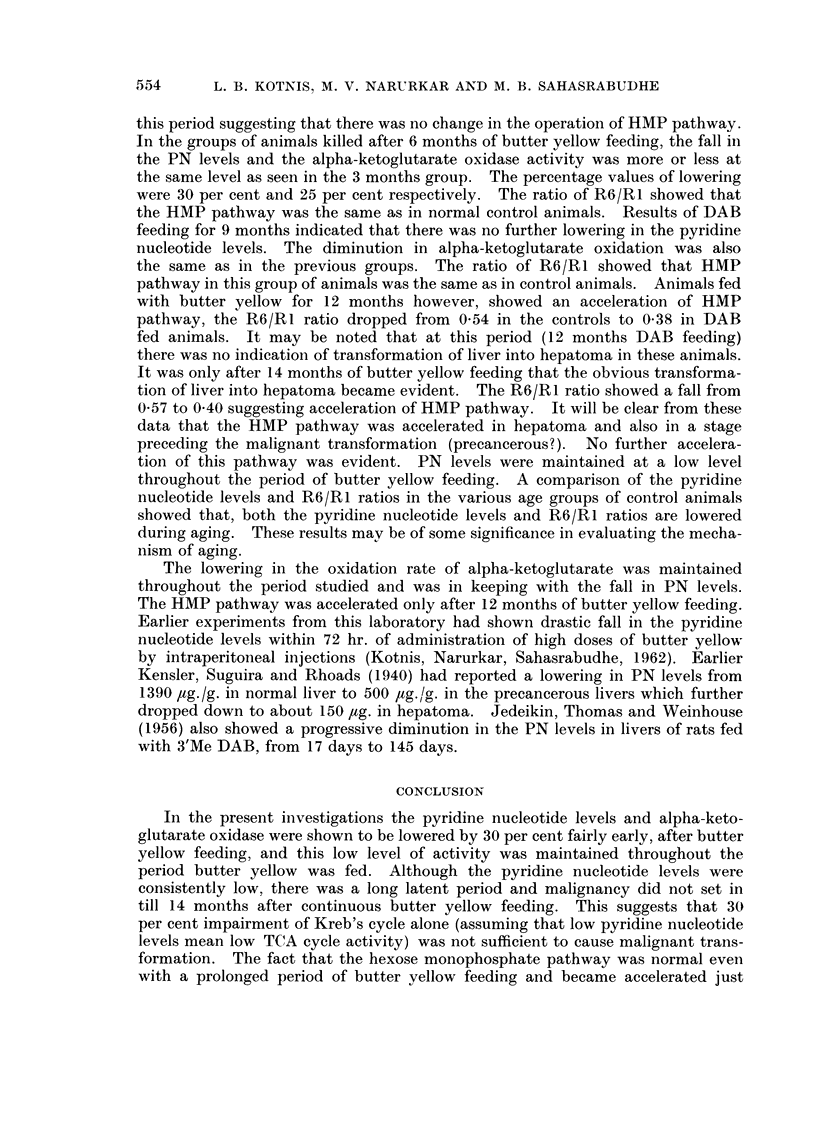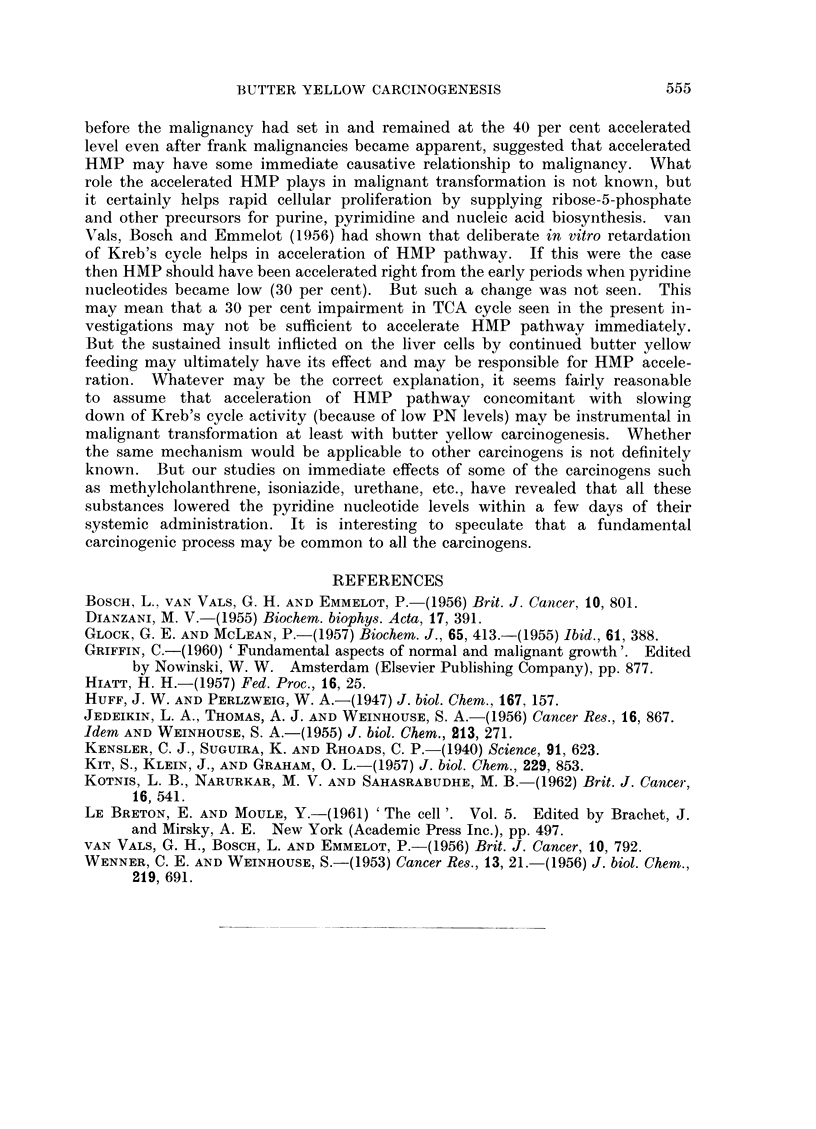# Role of Pyridine Nucleotides and Hexose Monophosphate Pathway in Butter Yellow (DAB) Carcinogenesis

**DOI:** 10.1038/bjc.1962.63

**Published:** 1962-09

**Authors:** L. B. Kotnis, M. V. Narurkar, M. B. Sahasrabudhe


### ROLE OF PYRIDINE NUCLEOTIDES AND HEXOSE MONOPHOSPHATE PATHWAY IN BUTTER YELLOW (DAB) CARCINOGENESIS

L. B. KOTNIS, M. V. NARURKAR AND M. B. SAHASRABUDHE

*From the Biology Division, Atomic Energy Establishment, Trombay, Bombay, India*

Received for publication July 5, 1962

In spite of sustained and concentrated efforts, the mechanism of carcinogenesis still remains ill-understood. Various hypotheses have been postulated but none of these seems to be universally applicable. This either means that cancer is a group of diseases and therefore the mechanism of their causation cannot be uniform, or, that our understanding of the disease is far from complete, thus necessitating postulation of different hypotheses to suit individual cases. Considerable information regarding the malignant growth has accumulated in recent years. A few quantitative differences between the levels and metabolic pathways of normal and malignant tissues have been discovered. Outstanding among these are (1) a rapid rate of nucleic acid synthesis (Le Breton and Moulé, 1961 ; Griffin, 1960) (2) low levels of pyridine nucleotides (PN)* (Glock and McLean, 1957; Jedeikin and Weinhouse, 1955), and (3) an acceleration of aerobic glycolysis or hexose monophosphate (HMP) pathway (Kit, Klein and Graham, 1957 ; Hiatt, 1957 ; van Vals, Bosch and Emmelot, 1956). Rapid nucleic acid synthesis seems to be in keeping with the demand to maintain increased cellular multiplication, but the significance of low pyridine nucleotide (PN) levels and accelerated HMP pathway does not appear to have been adequately investigated. It is necessary to know whether these biochemical changes have any role at all in the causation of malignant transformation or whether these are its effects. It is also necessary to know the stage at which these changes become evident during carcinogenesis. A detailed study of PN levels, PN-dependent alpha-ketoglutarate oxidation and the operation of HMP pathway at various intervals of time during butter yellow carcinogenesis, was therefore undertaken. Butter yellow or dimethylaminoazobenzene (DAB) was chosen for these studies. Since induction of hepatoma was a comparatively slow process, it afforded ample opportunities of studying the changes, if any, at various intervals of time. The second advantage in selecting butter yellow carcinogenesis for study was the availability of a well defined tissue (liver) as an appropriate control for comparison.

### MATERIALS AND METHODS

Two months old Wistar female rats obtained from our colony were put on butter yellow diet containing 0·06 per cent DAB. The other constituents were :

* The term " Pyridine nucleotides " or " PN " denotes the sum total of DPN, DPNH, TPN and TPNH.
" Reduced PN " denotes sum total of DPNH and TPNH.
" Oxidised PN " denotes sum total of DPN and TPN.

rice 76 per cent, casein 15 per cent, cod liver oil 2 per cent, olive oil 4 per cent and salt 4 per cent. The corresponding control animals were fed on the same diet but without the addition of azo dye. Groups of animals were killed at the intervals of $1\frac{1}{2}$–3, 6, 9, 12 and 15 months after the butter yellow feeding was started. In these experiments hepatoma became evident between 13–15 months after butter yellow feeding. Pyridine nucleotide levels, alpha-ketoglutarate oxidation and HMP pathway were determined in the livers of controls and butter yellow fed rats.

*Estimation of pyridine nucleotides*

The estimation of pyridine nucleotides was carried out according to the method of Huff and Perlzweig (1947) as modified by Dianzani (1955). This method is based on the formation of a fluorescent condensation product between pyridine nucleotides and acetone in presence of alkali. Briefly, the method consisted of homogenizing the tissue in 0·25 M sucrose containing 2 per cent nicotinamide. 10 ml. aliquots of 10 per cent homogenates were then deproteinized by addition of 1 ml. of 20 per cent trichloroacetic acid, immediately followed by the addition of 1 ml. of hydrogen peroxide (30 per cent) to oxidize the reduced nucleotides. The deproteinized extracts were then filtered through Whatman No. 1 filter paper and 1 ml. aliquots of the filtrates with appropriate dilutions were taken for developing fluorescence. Fluorescence was developed by adding 0·5 ml. of acetone and 0·2 ml. of 6 N NaOH followed by the addition of 0·3 ml. of 6 N HCl to neutralize the excess alkali. The tubes were heated in a water bath at 100° C. for 5 min., immediately chilled in ice and 1 ml. of 20 per cent $KH_2PO_4$ was added. The fluorescence thus developed was measured in a Pfaltz and Baur fluorimeter with bluish green filters.

*Determination of alpha-ketoglutarate oxidase*

The method employed for measuring oxidation of alpha-ketoglutarate was essentially the same as described by Wenner and Weinhouse (1953), except that the addition of DPN to reaction flasks was omitted. The medium contained in their final concentrations, $MgSO_4$ $6 \times 10^{-3}$ M, sodium fumarate $14 \times 10^{-5}$ M, ATP $4 \times 10^{-3}$ M, cytochrome C $8 \times 10^{-3}$ M, phosphate buffer (pH 7·4) $12 \times 10^{-3}$ M, KCl $13{\cdot}2 \times 10^{-2}$ M and substrate 0·012 M. The final volume of the incubation medium was 3·2 ml. inclusive of 1·0 ml. of the homogenate containing 100 mg. of wet tissue. The oxygen uptake was measured in conventional Warburg flasks for a period of 1 hr.

*Determination of the rate of HMP pathway*

Hexose monophosphate (HMP) pathway was evaluated by using glucose labelled with $^{14}C$ at the 1st and the 6th carbon atoms. The medium consisted of $MgSO_4$ $6 \times 10^{-3}$ M, potassium fumarate $14 \times 10^{-5}$ M, cytochrome C $8 \times 10^{-5}$ M, phosphate buffer (pH 7·4) $12 \times 10^{-3}$ M, KCl $2{\cdot}8 \times 10^{-1}$ M, DPN $4 \times 10^{-3}$ M. Glucose in the concentration of $4 \times 10^{-2}$ M was added as substrate (Wenner and Weinhouse, 1956). 0·1 $\mu$c of glucose-1-$^{14}C$ (or glucose-6-$^{14}C$ as the case may be) was added per flask. The central well contained filter paper strip soaked in 0·2 ml. of 10 per cent KOH to absorb the $CO_2$ evolved. 0·5 ml. of the homogenate containing 100 mg. of wet tissue was added to make the final volume 3·2 ml.

After 2½ hr. of incubation, the reaction was stopped by tipping 0·4 ml. of 4 N $H_2SO_4$. The shaking of Warburg flasks was continued for a further period of 20 min. to absorb any further $CO_2$ evolved. The contents of the central well of each flask were quantitatively transferred with hot water to a tube and 20 per cent of $BaCl_2$ along with carrier $Na_2CO_3$ was added. This procedure ensured complete precipitation of evolved radioactive $CO_2$ and $BaCO_3$. The $BaCO_3$ precipitates were collected on filter paper discs under vacuum filtration and washed free of $BaCl_2$. The filter paper discs were carefully transferred to planchets for measurements of radioactivity in a windowless gas flow counter.

### RESULTS AND DISCUSSION

Levels of PN at different intervals of time after butter yellow feeding are present in Table I. Tables II and III give the data on alpha-ketoglutarate

TABLE I.—*Levels of Pyridine Nucleotide in Livers of Rats Fed with p-Dimethylaminoazobenzene (DAB) at Various Intervals*

| Group | Age of animals (months) | Period of DAB feeding (months) | Pyridine Nucleotides μg./g. of wet tissue: Oxidized PN Control | Oxidized PN DAB fed | Oxidized PN % Fall | Reduced PN Control | Reduced PN DAB fed | Reduced PN % Fall | Total PN Control | Total PN DAB fed | Total PN % |
|---|---|---|---|---|---|---|---|---|---|---|---|
| I | 4–5 | 1½–3 | 433±26 | 345±32 | 20 | 385±35 | 265±32 | 31 | 818±38 | 610±18 | 25 |
| II | 8–10 | 6 | 565±14 | 400±32 | 29 | 312±43 | 211±43 | 32 | 876±44 | 611±40 | 30 |
| III | 11–13 | 9–10 | 439±7 | 315±9 | 25 | 379±5 | 259±7 | 31 | 800±27 | 574±15 | 28 |
| IV | 14–16 | 12 | 428±6 | 318±21 | 25 | 245±12 | 187±29 | 24 | 672±9 | 505±43 | 25 |
| V | 18–19 | 15–17 | 400±7 | 273±25 | 31 | 269±14 | 207±20 | 23 | 661±15 | 440±33 | 33 |

TABLE II.—*Alpha-ketoglutarate Oxidation by Liver Homogenates of Rats Fed with p-Dimethylaminoazobenzene at Various Intervals*

| Group | Age of animals (months) | Periods of feeding the diet (months) | Alpha-ketoglutarate oxidation μl. of $O_2$ uptake/100 mg. of wet liver: Control | DAB fed | % Fall |
|---|---|---|---|---|---|
| I | 4–5 females | 1½–3 | 127±8 | 93±3 | 27 |
| II | 8–10 females | 6 | 121±9 | 91±12 | 25 |
| III | 11–13 females | 9–10 | 129±8 | 99±3 | 23 |
| IV | 14–16 females | 12 | 125±6 | 88±4 | 30 |
| V | 18–19 females | 15–17 (Hepatoma) | 150±5 | 101±8 | 32 |

oxidase activity and operation of HMP pathway respectively. The overall data appears in Table IV. It will be seen that the PN levels were lowered by about 25 per cent as early as 1½ to 3 months after butter yellow feeding. The alpha-ketoglutarate oxidation which is DPN dependent was also reduced to about 27 per cent at this period. There was no change in the R6/R1 ratio at

TABLE III.—*Operation of Hexose Monophosphate Pathway in Liver Homogenates of Normal and DAB Fed Rats at Various Intervals*

| Group | Age of animals (months) | Period of feeding diet (months) | Experiment number | $^{14}CO_2$ activity, counted as $Ba^{14}CO_3$ in counts/minute: Controls G-1-$^{14}$C | Controls G-6-$^{14}$C | DAB fed G-1-$^{14}$C | DAB fed G-6-$^{14}$C | Ratio R6/R1 Control | Ratio R6/R1 DAB fed |
|---|---|---|---|---|---|---|---|---|---|
| I | 4–5 | 1½–3 | I | 7443 | 4638 | 6168 | 3849 | 0·62 | 0·62 |
| | | | II | 5009 | 3474 | 6372 | 3840 | 0·68 | 0·61 |
| | | | III | 8466 | 5138 | 6136 | 3675 | 0·60 | 0·61 |
| | | | IV | 7162 | 4297 | 7550 | 4532 | 0·60 | 0·60 |
| | | | V | 6091 | 3837 | 6583 | 4081 | 0·63 | 0·62 |
| | | | | | | | | 0·62±0·01 | 0·61±0·01 |
| II | 8–10 | 6 | I | 4477 | 2832 | 4010 | 2571 | 0·63 | 0·64 |
| | | | II | 4917 | 2832 | 3517 | 2142 | 0·58 | 0·61 |
| | | | III | 2321 | 1974 | 3605 | 2180 | 0·63 | 0·60 |
| | | | IV | 2658 | 1685 | 3605 | 2505 | 0·64 | 0·69 |
| | | | V | 2827 | 1825 | 4012 | 2826 | 0·65 | 0·69 |
| | | | | | | | | 0·62±0·01 | 0·64±0·02 |
| III | 11–12 | 9–10 | I | 6170 | 3579 | 7823 | 4533 | 0·58 | 0·57 |
| | | | II | 5097 | 3178 | 5745 | 3615 | 0·62 | 0·63 |
| | | | III | 6350 | 3756 | 7775 | 4504 | 0·59 | 0·57 |
| | | | IV | 5614 | 3458 | 8704 | 5094 | 0·61 | 0·58 |
| | | | | | | | | 0·60±0·01 | 0·61±0·02 |
| IV | 14–16 | 12 | I | 5047 | 2655 | 6672 | 2455 | 0·53 | 0·36 |
| | | | II | 4595 | 2589 | 6590 | 2914 | 0·56 | 0·44 |
| | | | III | 4185 | 2265 | 4987 | 1770 | 0·54 | 0·35 |
| | | | IV | 5595 | 3133 | 6059 | 2302 | 0·56 | 0·38 |
| | | | V | 4880 | 2635 | 6090 | 2192 | 0·54 | 0·36 |
| | | | | | | | | 0·54±0·01 | 0·38±0·01 |
| V | 18–19 | 15–17 | I | 2156 | 1248 | 3994 | 1279 | 0·57 | 0·34 |
| | | | II | 5763 | 3050 | 6620 | 2658 | 0·55 | 0·40 |
| | | | III | 1699 | 1015 | 1942 | 909 | 0·59 | 0·46 |
| | | | V | 2549 | 1524 | 2915 | 1202 | 0·59 | 0·40 |
| | | | | | | | | 0·57±0·01 | 0·40±0·014 |

TABLE IV.—*Levels of Pyridine Nucleotides, Alpha-ketoglutarate Oxidation and Operation of Hexose Monophosphate Pathway in Livers of Normal and DAB Fed Rats at Various Intervals*

| Group | Age of animals (months) | Period of DAB feeding (months) | Pyridine nucleotides µg./g. of wet liver weight: Control | DAB fed | % Fall | Alpha-ketoglutarate oxidation µl. of $O_2$ uptake/100 mg. wet weight: Control | DAB fed | % Fall | Hexose monophosphate Pathway R6/R1 ratio: Control | DAB fed |
|---|---|---|---|---|---|---|---|---|---|---|
| I | 4–5 | 1½–3 | 818±38 | 610±18 | 25 | 127±8 | 93±3 | 27 | 0·62±0·01 | 0·61±0·01 |
| II | 8–10 | 6 | 876±44 | 611±40 | 30 | 121±9 | 91±12 | 25 | 0·62±0·01 | 0·64±0·02 |
| III | 11–13 | 9–10 | 800±27 | 574±15 | 28 | 129±8 | 99±3 | 23 | 0·60±0·01 | 0·61±0·02 |
| IV | 14–16 | 12 | 672±9 | 505±43 | 25 | 125±6 | 88±4 | 30 | 0·54±0·01 | 0·38±0·01 |
| V | 18–19 | 15–17 (Hepatoma) | 661±15 | 440±33 | 33 | 150±5 | 101±8 | 32 | 0·57±0·01 | 0·40±0·014 |

this period suggesting that there was no change in the operation of HMP pathway. In the groups of animals killed after 6 months of butter yellow feeding, the fall in the PN levels and the alpha-ketoglutarate oxidase activity was more or less at the same level as seen in the 3 months group. The percentage values of lowering were 30 per cent and 25 per cent respectively. The ratio of R6/R1 showed that the HMP pathway was the same as in normal control animals. Results of DAB feeding for 9 months indicated that there was no further lowering in the pyridine nucleotide levels. The diminution in alpha-ketoglutarate oxidation was also the same as in the previous groups. The ratio of R6/R1 showed that HMP pathway in this group of animals was the same as in control animals. Animals fed with butter yellow for 12 months however, showed an acceleration of HMP pathway, the R6/R1 ratio dropped from 0·54 in the controls to 0·38 in DAB fed animals. It may be noted that at this period (12 months DAB feeding) there was no indication of transformation of liver into hepatoma in these animals. It was only after 14 months of butter yellow feeding that the obvious transformation of liver into hepatoma became evident. The R6/R1 ratio showed a fall from 0·57 to 0·40 suggesting acceleration of HMP pathway. It will be clear from these data that the HMP pathway was accelerated in hepatoma and also in a stage preceding the malignant transformation (precancerous?). No further acceleration of this pathway was evident. PN levels were maintained at a low level throughout the period of butter yellow feeding. A comparison of the pyridine nucleotide levels and R6/R1 ratios in the various age groups of control animals showed that, both the pyridine nucleotide levels and R6/R1 ratios are lowered during aging. These results may be of some significance in evaluating the mechanism of aging.

The lowering in the oxidation rate of alpha-ketoglutarate was maintained throughout the period studied and was in keeping with the fall in PN levels. The HMP pathway was accelerated only after 12 months of butter yellow feeding. Earlier experiments from this laboratory had shown drastic fall in the pyridine nucleotide levels within 72 hr. of administration of high doses of butter yellow by intraperitoneal injections (Kotnis, Narurkar, Sahasrabudhe, 1962). Earlier Kensler, Suguira and Rhoads (1940) had reported a lowering in PN levels from 1390 $\mu$g./g. in normal liver to 500 $\mu$g./g. in the precancerous livers which further dropped down to about 150 $\mu$g. in hepatoma. Jedeikin, Thomas and Weinhouse (1956) also showed a progressive diminution in the PN levels in livers of rats fed with 3′Me DAB, from 17 days to 145 days.

### CONCLUSION

In the present investigations the pyridine nucleotide levels and alpha-ketoglutarate oxidase were shown to be lowered by 30 per cent fairly early, after butter yellow feeding, and this low level of activity was maintained throughout the period butter yellow was fed. Although the pyridine nucleotide levels were consistently low, there was a long latent period and malignancy did not set in till 14 months after continuous butter yellow feeding. This suggests that 30 per cent impairment of Kreb's cycle alone (assuming that low pyridine nucleotide levels mean low TCA cycle activity) was not sufficient to cause malignant transformation. The fact that the hexose monophosphate pathway was normal even with a prolonged period of butter yellow feeding and became accelerated just before the malignancy had set in and remained at the 40 per cent accelerated level even after frank malignancies became apparent, suggested that accelerated HMP may have some immediate causative relationship to malignancy. What role the accelerated HMP plays in malignant transformation is not known, but it certainly helps rapid cellular proliferation by supplying ribose-5-phosphate and other precursors for purine, pyrimidine and nucleic acid biosynthesis. van Vals, Bosch and Emmelot (1956) had shown that deliberate *in vitro* retardation of Kreb's cycle helps in acceleration of HMP pathway. If this were the case then HMP should have been accelerated right from the early periods when pyridine nucleotides became low (30 per cent). But such a change was not seen. This may mean that a 30 per cent impairment in TCA cycle seen in the present investigations may not be sufficient to accelerate HMP pathway immediately. But the sustained insult inflicted on the liver cells by continued butter yellow feeding may ultimately have its effect and may be responsible for HMP acceleration. Whatever may be the correct explanation, it seems fairly reasonable to assume that acceleration of HMP pathway concomitant with slowing down of Kreb's cycle activity (because of low PN levels) may be instrumental in malignant transformation at least with butter yellow carcinogenesis. Whether the same mechanism would be applicable to other carcinogens is not definitely known. But our studies on immediate effects of some of the carcinogens such as methylcholanthrene, isoniazide, urethane, etc., have revealed that all these substances lowered the pyridine nucleotide levels within a few days of their systemic administration. It is interesting to speculate that a fundamental carcinogenic process may be common to all the carcinogens.

### REFERENCES

Bosch, L., van Vals, G. H. and Emmelot, P.—(1956) *Brit. J. Cancer*, **10**, 801.

Dianzani, M. V.—(1955) *Biochem. biophys. Acta*, **17**, 391.

Glock, G. E. and McLean, P.—(1957) *Biochem. J.*, **65**, 413.—(1955) *Ibid.*, **61**, 388.

Griffin, C.—(1960) ' Fundamental aspects of normal and malignant growth '. Edited by Nowinski, W. W. Amsterdam (Elsevier Publishing Company), pp. 877.

Hiatt, H. H.—(1957) *Fed. Proc.*, **16**, 25.

Huff, J. W. and Perlzweig, W. A.—(1947) *J. biol. Chem.*, **167**, 157.

Jedeikin, L. A., Thomas, A. J. and Weinhouse, S. A.—(1956) *Cancer Res.*, **16**, 867.

*Idem* and Weinhouse, S. A.—(1955) *J. biol. Chem.*, **213**, 271.

Kensler, C. J., Suguira, K. and Rhoads, C. P.—(1940) *Science*, **91**, 623.

Kit, S., Klein, J., and Graham, O. L.—(1957) *J. biol. Chem.*, **229**, 853.

Kotnis, L. B., Narurkar, M. V. and Sahasrabudhe, M. B.—(1962) *Brit. J. Cancer*, **16**, 541.

Le Breton, E. and Moule, Y.—(1961) ' The cell '. Vol. 5. Edited by Brachet, J. and Mirsky, A. E. New York (Academic Press Inc.), pp. 497.

van Vals, G. H., Bosch, L. and Emmelot, P.—(1956) *Brit. J. Cancer*, **10**, 792.

Wenner, C. E. and Weinhouse, S.—(1953) *Cancer Res.*, **13**, 21.—(1956) *J. biol. Chem.*, **219**, 691.